# Complementary Parent Components for Pediatric Pain Families: Innovations in Treatment

**DOI:** 10.3390/children7010004

**Published:** 2020-01-01

**Authors:** Beth S. Russell, Jessica W. Guite, Kendra J. Homan, Rebecca M. Tepe, Sara E. Williams

**Affiliations:** 1 Human Development & Family Science, The University of Connecticut, 380-398 Mansfield Rd, Storrs, CT 06269, USA; 2 School of Nursing, University of Connecticut, 231 Glenbrook Rd., Unit 4026, Storrs, CT 06269, USA; jessica.guite@uconn.edu; 3 School of Medicine, University of Connecticut, 200 Academic Way, Farmington, CT 06032, USA; 4 Department of Pediatrics, University of Cincinnati College of Medicine, 331 Albert Sabin Way, Cincinnati, OH 45229, USA; Kendra.Homan@cchmc.org (K.J.H.); sara.williams@cchmc.org (S.E.W.); 5 Division of Behavioral Medicine and Clinical Psychology, Cincinnati Children’s Hospital Medical Center, 3333 Burnet Ave, Cincinnati, OH 45229, USA; 6 Division of Social Services, Cincinnati Children’s Hospital Medical Center, 3333 Burnet Ave, Cincinnati, OH 45229, USA; rebecca.tepe@cchmc.org

**Keywords:** chronic pain, parenting intervention, intensive interdisciplinary pain treatment

## Abstract

For families with a child with chronic pain, the home environment is the context in which adaptive or maladaptive illness behaviors are developed. Supporting families to effectively cope with their child’s chronic pain is a critical need. This work analyzes intervention approaches from emerging treatment programs to support families coping with pediatric pain that diverge from traditional treatment models by specifically targeting parents. Two novel parent intervention programs are presented that consider caregiver needs in both outpatient and inpatient pain treatment settings: *Parents as Coping Coaches* and *Putting Parents FIRST*. These programs are evaluated through comparing parental training components across different stages of treatment. Additionally, the efficacy of *Putting Parents FIRST* in promoting maintenance of children’s functional gains achieved in intensive interdisciplinary pain treatment is presented, and compared to previous results of the efficacy of *Putting Parents FIRST*. Specifically, outcomes of 36 children whose parents received the intervention in *Putting Parents FIRST* were compared to a matched control sample of children whose parents did not receive the parent intervention. Similar to the findings from *Parents as Coping Coaches*, results indicated that patients whose parents received the intervention maintained/improved program gains in disability, coping, and pain significantly more than patients whose parents did not receive the intervention. Implications for parent-focused intervention development efforts targeting parent and youth functioning in the context of pediatric chronic pain are considered.

## 1. Introduction

For families with a child with a chronic pain condition, the routines and relationships of the home are the context in which family members develop skills to adapt to the diagnosis and its associated symptoms, but also may reinforce maladaptive illness behaviors [1]. Supporting children and families to cope with chronic pain effectively is a critical need made even more important amidst rising healthcare costs that may limit access to care [1,2]. There has been an 831% increase in hospital admissions for pediatric pain complaints from 2004 to 2010 [3], with the cost of this care gaining more attention within the current era of healthcare reform under way in the United States in particular. Notably, a 2014 estimate of total cost to the American society for adolescents with “moderate to severe” chronic pain was extrapolated to $19.5 billion annually, primarily resulting from direct medical costs and productivity losses, with parents assuming the majority of this burden [4].

An aspect of this problem that has received less research attention to date is what particular parent and family factors in pediatric chronic pain contribute to seeking treatment and service utilization. Existing research suggests that parental chronic pain, distress, overly protective parental behaviors, and parental pain catastrophizing may all be potential factors that contribute to patterns of healthcare utilization for children with chronic pain [5,6,7,8]. There is also evidence to suggest that parents tend to struggle to understand chronic pain symptoms and the best way to manage their children’s highly distressing pain complaints [9,10,11]. Chronic pain is distinct from an acute experience when pain symptoms last longer than 3 months and negatively impacts activity; in children, chronic pain most commonly presents as abdominal pain, headache, and musculoskeletal pain. A main tenet of chronic pain intervention is that function improves before pain, indicating that children have to cope with the pain and move through it *before* achieving pain reduction. This approach often feels counterintuitive to families because the natural response to children’s typical/acute pain experiences is increased caretaking (e.g., frequent asking about pain, attending to pain behaviors, reducing expectation for normal functioning until pain subsides, accommodating the pain). However, in the case of chronic pain, these otherwise normal parenting behaviors only make the chronic pain condition worse in the long term by reinforcing the pain and disability cycle [10]. Therefore, parents may find it difficult to enact the treatment plans recommended for their children’s chronic pain and also may find it distressing as having to ask children to push through pain. This distress can result in seeking treatment in the emergency department—which is an acute treatment setting that generally neither resolves nor typically mitigates chronic problems—and pursuing what may inadvertently become excessive and often inconclusive consultation from specialists and/or diagnostic testing [12].

Opportunities to support the needs of families coping with pediatric pain that diverge from traditional treatment models (which are typically patient focused) are needed to comprehensively address this problem. Research on intensive interdisciplinary pain treatment (IIPT) examining the effects of including parents in children’s treatment found that parents made significant improvements in their parenting and emotional responses. Specifically, parents made reductions in protective parenting responses (e.g., more encouragement of normal activity) [13], in their own emotional status and coping efforts (e.g., reduction in depressive symptoms less pain catastrophizing, and improved psychological flexibility) [14,15], and decreased protective, monitoring, and minimizing responses during treatment (e.g., fewer instances of checking in about symptoms, less attention to pain behaviors, more recognition of children’s positive behaviors) [16]. As a result, more pain-focused outpatient and inpatient programs are developing modules/intervention to target parents of children with chronic pain to similarly positively influence child outcomes. Such interventions have demonstrated positive changes in parents’ psychological flexibility in parents of children with chronic pain [17] and improvements in mental health, parenting behaviors, health status, and problem-solving skills [18]. To date no research has examined the effects of a parent program on patient outcomes, though preliminary evidence indicates this is a promising line of inquiry [19]. Recently, Guite and colleagues [19] published evidence that parent-focused interventions for this population led to the decrease in caregiving burden, protective and monitoring parenting responses to the adolescent’s pain, and parent-perceived adolescent pain burden and disability. They further note positive associations between parent and adolescent reports of distress tolerance and readiness to change observed both pre- and post-intervention. To advance this work, process evaluations that address the *how* of intervention implementation (e.g., assessing elements of treatment feasibility and acceptability rather than solely its outcomes) are crucial for development of treatment innovations, as they play a vital role in describing potential barriers associated with delivering treatment across varying treatment settings—in this case to parents of children with chronic pain receiving support through outpatient and inpatient programming.

The current paper describes such an effort, including summaries of two complementary intervention programs for parents of children of chronic pain, delivered at different points in this treatment setting continuum: *Parents as Coping Coaches* (*PaCC*), for parents of youth receiving treatment in a multi-disciplinary outpatient setting [19,20], and *Putting Parents FIRST*, for parents of youth receiving treatment in an inpatient IIPT setting. Based on our previous collaborations across these programs, first, we provide a detailed comparison of parental training components of *PaCC,* as a previously published outpatient intervention, to those of *Putting Parents FIRST,* as an emerging model for inpatient intervention. Then, to compare with published efficacy results from *PaCC* [19,20] and advance the discourse on patient impacts over time, we present initial longitudinal program outcomes from the *Putting Parents FIRST*. Specifically, we hypothesized that children whose parents received the *Putting Parents FIRST* intervention would maintain program gains more effectively than children whose parents did not receive the intervention.

## 2. Methods

### 2.1. Parents as Coping Coaches

#### Treatment

*Parents as Coping Coaches* (*PaCC*) is a recently developed brief group intervention that balances time away from family with parent-focused time to attend to self-care and problem-solving skills, in addition to receiving peer social support. Parents (of adolescents between the age 12 and 18 years (M = 15.2; 68% female) were recruited to participate in this Institutional Review Board (IRB) approved protocol (#15-088) through an outpatient pediatric pain clinic. The initial pilot study intervention groups were facilitated by a social worker and marriage and family therapist, with 3–6 parents in attendance. After giving consent, parents attend three 120-minute consecutive weekly sessions that cover three content domains: (1) Pain education material which reviews the physiology of pain, the gate control theory of pain, and miscarried helping; (2) parent-adolescent communication content focuses on joint problem solving through active listening and promoting developmentally appropriate levels of autonomy to encourage adolescents’ self-management of pain; (3) coping skills information focuses on distress tolerance skill building and self-care through mindfulness and other coping skill approaches. These content domains are evident in each of the three sessions, with the coping skills domain comprising the largest content proportion overall. Parent, adolescent, and parent-adolescent dyad impacts from *PaCC* have been published previously [19,20,21], and indicate significant pre- to post-intervention differences in authoritarian parenting (authoritarian subscale of the parenting styles and dimensions questionnaire, *t*(19) = 2.70, *p* < 0.05); caregiver burden (bath adolescent pain parent impact questionnaire sub scales including: depression, *t*(20) = 2.53, *p* = 0.020; anxiety *t*(20) = 3.13, *p* = 0.005; self-blame/helpfulness, *t*(20) = 3.15, *p* = 0.005; and parental behavior *t*(20) = 4.86, *p* = 0.001); parent responses to their adolescent’s pain (adult responses to children’s symptoms subscales including: protect, *t*(20) = 2.27, *p* = 0.034 and monitor, *t*(20) = 5.46, *p* = 0.001); parent perceptions of pain burden (pain burden inventory/sickle cell pain burden interview, *t*(20) = 3.43, *p* = 0.003); and perceptions of adolescent functional disability (functional disability inventory, *t*(20) = 2.82, *p* = 0.11) [22,23,24,25,26,27]. Importantly, the *PaCC* project was the first to publish associations between parent and adolescent reports of and readiness to change from pre- to post-intervention [19]: Pre-intervention, parent and adolescent reports of readiness to change were significantly correlated on the precontemplation subscale (*r* = 0.54, *p* < 0.01) as were parent and adolescent reports of pain burden (*r* = 0.56, *p* < 0.01). At post-intervention, however, parent reports for the precontemplation subscale were negatively correlated with adolescent reports on the contemplation and action/maintenance subscales (*r* = 0.67, *p* < 0.01; *r* = 0.55, *p* < 0.05 respectively). These preliminary findings may indicate adolescent patients’ greater engagement in a pain self-management approach could be related to parents’ participation in *PaCC*. These results demonstrate the strengths of the program in leading to significant parent and child outcomes, and situate it as a valid comparator for the initial findings from the *Putting Parents FIRST* intervention presented below.

### 2.2. Putting Parents FIRST

#### 2.2.1. Treatment

*Putting Parents FIRST* is a group-based parent intervention that is delivered in an inpatient pediatric IIPT setting to help parents learn how to support children with severe levels of pain and disability both during their treatment and in preparation for going home. The intervention is co-facilitated by a social worker and psychologist, with 2–3 parents meeting per group. Parents attend three 60-minute consecutive weekly sessions that cover three content domains: (1) Pain education material teaches parents about the neuroscience of chronic pain (including the gate control theory of pain and the pain and disability cycle), which underscores the rationale for a functional restoration approach; (2) parenting content focuses on addressing parental guilt, identifying parenting styles, and learning how to apply an authoritative parenting approach in the context of chronic pain; (3) transition material provides parents with skill-building to manage children’s chronic pain at home, including setting functional expectations, communicating effectively, and implementing positive reinforcement and consequence plans to support children in maintaining treatment gains. These content domains are specific to each session, and parents receive all three sessions in a rotating fashion during their child’s admission to the IIPT program.

#### 2.2.2. Participants

From the *Putting Parents FIRST* program, outcome data from a target sample of 36 pediatric patients who consecutively completed the IIPT program were included in the analysis. Admission criteria for the FIRST program include failure to progress in outpatient care (medical, psychological, and physical therapy) or lack of access to appropriate outpatient care, confirmed chronic pain diagnosis that is ongoing and lasting more than 6 months (including completion of all medical testing), and patient age between 9 and 19 years. For each of the pediatric patients in the target sample, an age (±12 months for females, ±24 for males), gender, and diagnosis matched patient enrolled in the IIPT program, but prior to the implementation of the *Putting Parents FIRST* intervention, was selected for comparison. The mean age of the total sample was 14.82 years (*SD* = 2.22); target group: *M* age = 14.78, *SD* = 2.40; comparison group: *M* age = 14.86, *SD* = 2.06. The majority of the patients were female (*n* = 50, 69%). Patients were predominantly Caucasian (*n* = 67, 93%) followed by African American (*n* = 3, 4%), Asian (*n* = 1, 1.5%), and Hispanic (*n* = 1, 1.5%). On average, group size was 2.5 parents per session. There was no loss of attendance in the *Putting Parents FIRST* program. Primary pain diagnoses were as follows: joint hypermobility syndromes (*n* = 28), amplified musculoskeletal pain syndrome (*n* = 16), complex regional pain syndrome (*n* = 10), abdominal pain (*n* = 8), musculoskeletal pain (*n* = 6), and headache (*n* = 4). Overall, in the IIPT program, on average about 25% of the patients have medical comorbidities (most commonly sleep, autonomic, and gastrointestinal disorders) and 70% have psychiatric comorbidities (most commonly anxiety, depressive, and neurodevelopmental disorder).

#### 2.2.3. Procedure

This study was approved by the Institutional Review Board of Cincinnati Children Hospital and Medical Center (IRB approval #2015-8104); consent was waived for review of de-identified electronic medical record data. A matched sample analysis (age, gender, diagnosis) was conducted comparing 36 children who completed the IIPT program *prior* to the addition of the parent-focused intervention, and 36 children who completed the IIPT program after the addition *and* whose parents received the new parent-focused intervention. Changes from admission to discharge and from discharge to one-month follow-up were examined using three patient self-report outcomes administered at those three time points: (1) functional disability inventory (FDI), (2) pain coping questionnaire (PCQ), and (3) numeric rating scale-11 (NRS-11). The FDI is a 15-item self-report inventory assessing children and adolescents’ perceived difficulty in the performance of daily activities in home, school, recreational, and social domains; higher scores indicate more functional disability [22]. The PCQ is a 3-item measure assessing children and adolescents’ ability to emotionally manage pain; lower scores indicate poorer coping [23]. The NRS-11 is a standardized numerical pain assessment rating; higher scores indicate greater pain intensity. For the purpose of this study, the average pain levels were rated [24].

## 3. Results

### 3.1. Comparison of Parental Training Components across Treatment Settings

A commonality to both *PaCC* and *Putting Parents FIRST* group-based programs is that they both include a session on pain education. In both programs, pain education provides a rationale for why a functional restoration model works for patients with chronic pain (e.g., gate control theory of pain, neurological understanding of pain mechanisms, the chronic pain and disability cycle, role of functional restoration in retraining the processing of pain in the brain). Each program tailored the material to meet parents where they are with consideration of the treatment setting. More specifically, *Putting Parents FIRST* examples focus on how material applies to more significant pain and disability given the inpatient IIPT setting, whereas as *PaCC* examples focus on solving everyday challenges that arise for families maintaining treatment schedules within a daily routine consistent with an outpatient treatment model. In this session, both *PaCC* and *Putting Parents FIRST* provide guidelines to parents on how to support children with chronic pain while also encouraging function (e.g., reduced attention for pain talk and behaviors, increased expectations for normal functioning despite pain, fewer family accommodations for functional disability, encouragement to remain active despite pain). These pain education components are believed to be integral to receiving parental buy-in and partnering with parents to approach their child’s symptoms in a new way.

The second sessions of *PaCC* and *Putting Parents FIRST* are somewhat different. *PaCC* primarily focuses on parent-adolescent communication, including normative patterns of communication during this developmental period. The session includes both didactic and role-playing opportunities to articulate ways parents can support their adolescent to problem solve effectively while promoting developmentally appropriate levels of adolescent autonomy in the process. Specifically, parents are taught how to lead their child through conversations about how a given problem might arise and how the parent and child can work together to take differing levels of responsibility for solving the dilemma, depending on the problem at hand. For example, different precipitating elements and needed actions are at play when the problem is completing chores at home versus establishing adaptations for school (that may require advocacy with teachers or administrators), and as such, parents and their adolescents have different roles and responsibilities in resolving each problem. Teaching parents this process empowers them to lead their child through a problem-solving approach, instead of solving problems for them, by encouraging parents to use active listening strategies to invite their adolescent’s participation at developmentally appropriate levels of ownership toward generating a solution. In this way, parents are given direct instruction and opportunities to practice providing support to their children, while empowering them to become more adaptive to coping with pain. In addition, practice of mindfulness training and distress tolerance skill building is also woven into this session, to help manage the common frustrations and negative emotions that parents experience when facing problems with their adolescents. Parents are encouraged to practice these skills over the week between *PaCC* group sessions, with a structured daily diary to remind them to practice the self-care skills. The over-arching goal for this session is to help parents use self-care skills during potentially tense and difficult interactions with their adolescent so that parents feel a sense of competence in managing their own negative emotions, while also supporting their child’s developmental needs for individuation.

Within the *Putting Parents FIRST* intervention, the emphasis of the second session is parenting a child with chronic pain, addressing parent guilt, identifying existing parenting styles, learning how to apply an authoritative parenting style to parenting a child with chronic pain, and engaging in parental self-care. Addressing parental guilt and evaluating parenting styles are two critical elements of this session. Regarding parental guilt, once enrolled in an IIPT setting that parents see is benefitting their child, many parents share a sense of guilt that they did not take a functional approach sooner. This session specifically addresses these challenges by normalizing parents’ feelings, validating their sense of responsibility, educating them on the paradoxical aspects of chronic pain management (e.g., that in order to help children improve their chronic pain they have to function through it), and helping parents begin to take care of themselves again, something that most have put off in favor of caring for their child. Regarding parenting styles, many parents of children with chronic pain fall into a cycle of permissive parenting practices as a result of confusion and frustration over managing their child’s pain. This often results in parents having few functional expectations for their child, such as allowing their child to miss school or family activities during a pain episode. While this parenting style makes sense in the short term (or for children who are acutely ill), when permissive parenting is employed over time for children with chronic conditions, this approach can accidentally reinforce a pain/disability cycle. To help parents understand their parenting style and how this influences the management of their child’s pain and function, parents complete a self-assessment and identify their parenting style out of the four types (permissive, authoritarian, authoritative, or uninvolved). The self-assessment intervention helps parents identify behaviors that they may need to change in order to implement authoritative parenting practices over permissive practices. The authoritative parenting style is characterized by having open communication with the child regarding expectations and consequences in a supportive, nurturing environment, which is the most effective parenting style to foster independence and self-regulation in children in general, and in the context of pain [25,26].

The third and final sessions of *PaCC* and *Putting Parents FIRST* also varied in content. *PaCC* emphasizes parental coping skills through distress tolerance skill building and self-care techniques introduced in the first two weeks of *PaCC*. During the third session, each of these activities are reviewed in light of parents’ experiences implementing these skills over the preceding 2 weeks. Parents are encouraged to reflect on their use of these skills and share examples of their use of problem-solving skills with their adolescent. Parents provide peer support and suggestions, facilitated by the group leader. Facilitators are careful to solicit and reinforce the positive experiences parents share with the group, and to model brainstorming alternative strategies when narratives are dominated by struggle—always deliberately offering validation and encouragement for facing obstacles in the future. The group includes positive reinforcement for parents’ continued engagement in the adolescent’s recommended treatment plan and also reinforces taking time to care for themselves and utilize the coping skills presented in group.

The final session of *Putting Parents FIRST* focuses on transition home after IIPT completion. Education on behavioral mechanisms to support long-lasting change in the home setting is provided, in addition to communication skill building similar to content delivered in the second session of *PaCC*. Specifically, parents are provided instruction on the functional expectations to have of their child at home (e.g., not talking about pain, attending all scheduled events, completing household chores, etc.) and how to create a schedule of daily activities that mirrors the schedule children follow in IIPT. Finally, parents learn how to complete a behavior contract regarding these functional expectations, including implementation of a reinforcement and/or consequences plan for not meeting functional expectations. Transitioning home after IIPT while maintaining and increasing the level of functioning gained while hospitalized is the goal of this treatment modality. This session was specifically created to help support parents in making individual and family changes so that children remain functional in the home environment. Feedback from parents who have received this instruction is that they find these tools (particularly the schedule and behavior contract provided in the program) useful to help them continue to promote functional expectations after discharge home.

### 3.2. Putting Parents FIRST Program Characteristics Associated with Significant Outcomes

**Figure 1:**
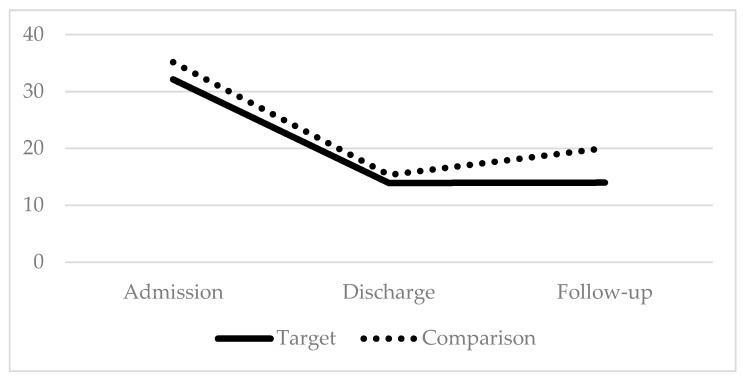
Functional Disability; Lower scores indicate improvement.

**Figure 2:**
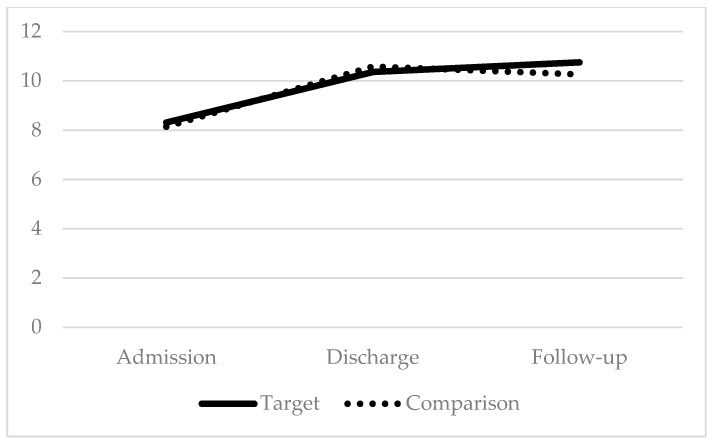
Coping: higher scores indicate improvement.

**Figure 3:**
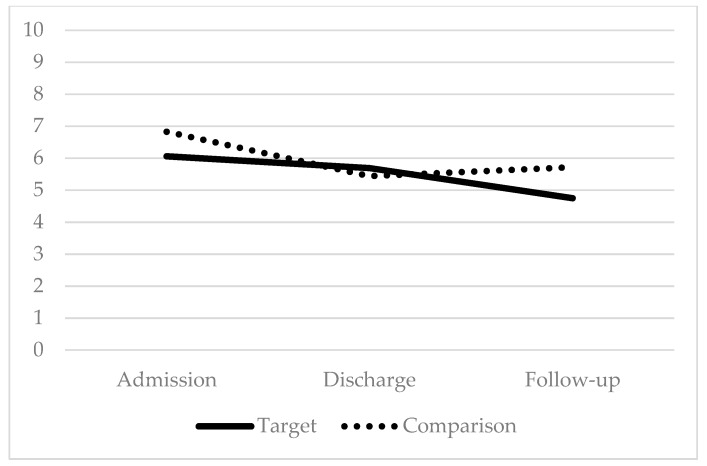
Average pain intensity: lower scores indicate improvement.

Paired t-tests were conducted to evaluate the efficacy of this parent training component in promoting maintenance of children’s functional gains after discharge from IIPT by comparing outcome measures of 36 children who completed the IIPT program *and* whose parents received the intervention (target group) to a matched control sample of 36 children who completed the IIPT program *prior* to the additional implementation of the parent intervention (comparison group). Results indicated that patients in both groups made similar gains during the program on disability and coping (*p* > 0.05); additionally, patients in the comparison group saw greater change in pain than patients in the target group, *t*(70) = −2.91, *p* < 0.01. However, at follow-up, patients in the target group maintained program gains for disability, coping, and pain and demonstrated significantly greater improvement than patients in the comparison group did for disability *t*(70) = 2.24, *p* < 0.05, coping *t*(68) = 2.13, *p* < 0.05, and pain *t*(70) = 2.56, *p* < 0.05 (see Figure 1, Figure 2 and Figure 3).

## 4. Discussion

Comparison of the content of two parent-focused interventions presented here evidenced similar overall structures with variability across setting, as well as differential maintenance of treatment gains in children whose parents had received intervention in *Putting Parents FIRST*, similar to previously published results of *PaCC*. Specifically, both parent interventions included information about pain education, parenting a child with chronic pain, and communication. While both interventions started with pain education, they differed in the order of delivery of information. Content differed slightly by treatment setting, including a focus on training parents in coping strategies in the outpatient group versus a focus on behavioral management strategies in the inpatient group. However, as noted from previously published data, both programs were found to be satisfactory and beneficial by parents [19,21]. While children’s clinical success was previously reported in the *PaCC* program [19,21], consistent with our hypothesis, results of this study also demonstrated success of the *Putting Parents FIRST* program in children’s significant maintenance of gains disability, coping, and pain among children whose parents received the intervention versus children whose parents did not. Taken together with previous findings, parent intervention in both outpatient and inpatient settings is effective in supporting children’s gains when receiving treatment for chronic pain.

These preliminary research efforts draw upon valuable initial implementation experience in real-life clinical contexts which can further inform the key process indicators (e.g., timing, dose, setting) that other parent-focused programs can consider. Specifically, intervention design efforts for this field must take great care to document and respond to family-centered needs. Mixed methods approaches to program evaluations that conduct both content and outcomes comparisons, as done in this study, can also provide valuable insight into how to shape and further inform the process of intervention design and implementation. In the case of pediatric chronic pain, interventions must address parents’ needs as a necessary co-occurring treatment, given the crucial role parents play in the family system and as gatekeepers to accessing treatment. Parents and their behavior provide the context of care for children, wherein the influence of parent behavior shifts as children age and approach developmentally appropriate individuation to increase their use of self-care skills [20,27,28,29]. Those with chronic health conditions likely see a delay in this process, with recent qualitative research suggesting the experience of chronic pediatric pain supports themes that include both enhanced and delayed developmental trajectories for adolescents coping with this condition [30]. As a result, a shared goal of interventionists is to identify the best content to provide parents at a given point in treatment flow and that affords the most intervention penetration, considering the strains parents can encounter caring for a child with chronic pain (e.g., physical and mental health challenges). Specifically, as a field we strive to implement parent interventions that reduce intrusive and anxious parenting and manage parental guilt and anxiety. Limiting the burden that parents can experience while supporting their children is another important goal with respect to limitations on time, finances, and access to care. We seek to reframe care recommendations into helpful, developmentally-appropriate, parenting goals rather than recommendations that may inadvertently encourage anxious parenting behaviors that can lead to dysfunctional family dynamics over time (i.e., emotional fusion) [30]. Within this pediatric pain population we face the question of which parent-focused elements seem consistently acceptable and effective, versus specific to, a particular moment in time.

Important intervention implementation considerations exist with respect to moving toward a more preventative orientation to pediatric pain that includes the timing of delivery. The increasing integration of behavioral health support in primary care settings, suggests that there are more opportunities along the way to intervene earlier and by doing so we may help to prevent symptoms becoming more chronic. Salamon and Cullinan recently proposed a preventative model of pediatric chronic pain that will be a critically important blueprint for developing lower cost and less time intensive approaches to treatment [31]. *PaCC*’s parent-focus on providing critical pain education, communication and emotion regulation skills—as well as both direct peer and interventionist delivered support for parents—may make it an optimal, preventative, lower-intensity intervention able to be delivered in a primary care setting or in a multidisciplinary outpatient pain treatment setting. At the other end of the care continuum, for families whose children reach high levels of pain and disability, the *Putting Parents FIRST* model is well-suited to support parents during admission and potentially improve translation of IIPT outcomes to the home setting. Future efforts to include parental intervention in any specific treatment setting should also include evaluation of cost savings and health care utilization with respect to burden on families to better understand their impact and potential benefit. As parent-focused treatment are disseminated more widely, and with better resources to enable increasingly robust methodologies (e.g., randomization in multi-site experimental trials), interventionists will be able to provide subgroup analyses based on care setting.

While including parental intervention alongside traditional patient-focused treatment in the field of pediatric pain intervention has many potential benefits, there are also limitations and considerations for implementation. In current clinical practice, intervention delivery may be limited by available resources and health care referral patterns. Specifically, while primary care or an early, integrated, approach may be most desirable from the standpoint of: (a) preventing chronicity of symptoms and patterns of disability and, (b) delivering less intensive (and costly) interventions, this approach is dependent on availability of access to behavioral health support in primary care or community settings. In addition, the patient and parent may not have experienced symptoms that are at a level that would sufficiently motivate a parent to engage in a 3-session intervention like *PaCC*, with respect the other competing time demands that families often juggle. It is certainly desirable to decrease the distress that accompanies a family’s decision to consult the ED for chronic pain symptoms, or to pursue inpatient treatment, from both a treatment intensity and cost standpoint. However, families seen in an acute or tertiary care setting may perhaps be more motivated to engage in behavioral treatment approaches as a result of the perceived acuity of the adolescent’s symptoms and disruption to family life. The inpatient setting is clearly the optimal setting for the delivery of the *Putting Parents FIRST* intervention. However, for an intervention like *PaCC*, which engages parents directly without their child’s direct involvement, the optimal timing may be at the point of agreement between the family and provider that a persistent pain problem exists and discussion of referral from primary to secondary (specialty) care. At this transition point, parents and patients acknowledge a need for additional intervention and may be optimally motivated to engage in behavioral changes and the recommendations in a less intensive, lower cost, outpatient setting. While many excellent options are increasingly available for families to utilize web-based and/or mobile phone interventions for support of pediatric chronic pain symptoms [32,33,34], these tech-based options may not be the best fit for all families. For example, while a positive aspect of these interventions is that they can help to remove barriers to access, a recent app-based intervention directed specifically at parents reported only small to medium effects for parent behavior outcomes [33]. In addition, these authors also note that parents’ feedback and recommendations, “highlighted a need for more opportunities to interact with other parents and to customize the content” [33], which is a task that interventions like *PaCC* or *Putting Parents FIRST* are well suited to accomplish. Future research is needed to better understand and test the optimal timing and intensity of intervention with the goal of preventing increasingly chronic and disabling symptoms for the child and accompanying parenting distress and disruption in family life.

## 5. Conclusions

Intervention strategies that support parents, who are both the gatekeepers for their child’s healthcare access and partners in providing ongoing support for their child, are proving to be an important consideration in both outpatient and inpatient pain treatment settings. The field is faced with compelling questions about the format and timing of these resources. This includes considerations about whether content should focus primarily on psychoeducation or skill-building approaches, with evidence available for the efficacy of both described. Treatment providers struggle to meet the needs of parents whose children present for treatment for pediatric chronic pain in various settings. As presented in this paper, parent intervention programs delivered at both the outpatient and inpatient setting are equally well suited to effectively provide parent training components specific to pain treatment. Both the Parents *and Coping Coaches (PaCC)* and *Putting Parents FIRST* interventions described are associated with positive outcomes among children and parents alike. Future studies should now consider the timing of the delivery of these parent-inclusive interventions, including whether parent intervention could align with preventative health models, along with tailoring content to appropriately support the needs of parents based on the severity of their child’s symptoms. Research indicates families can face high degrees of caregiver burden, in part contributed to by time consuming treatment coordination [10,19,21]. This presents care providers with both the challenge and the opportunity to help alleviate parent burden at multiple points along the spectrum of care. We offer these two novel interventions as two promising options for addressing this important unmet need.

## References

[1] Groenewald C.B., Palermo T.M. (2015). The price of pain: The economics of chronic adolescent pain. Pain Manag..

[2] Groenewald C.B., Wright D.R., Palermo T.M. (2015). Health care expenditures associated with pediatric pain-related conditions in the United States. Pain.

[3] Coffelt T.A., Bauer B.D., Carroll A.E. (2013). Inpatient characteristics of the child admitted with chronic pain. Pediatrics.

[4] Groenewald C.B., Essner B.S., Wright D., Fesinmeyer M.D., Palermo T.M. (2014). The economic costs of chronic pain among a cohort of treatment-seeking adolescents in the United States. J. Pain.

[5] Chow E.T., Otis J.D., Simons L.E. (2016). The Longitudinal Impact of Parent Distress and Behavior on Functional Outcomes Among Youth With Chronic Pain. J. Pain.

[6] Poppert Cordts K.M., Stone A.L., Beveridge J.K., Wilson A.C., Noel M. (2019). The (Parental) Whole Is Greater Than the Sum of Its Parts: A Multifactorial Model of Parent Factors in Pediatric Chronic Pain. J. Pain.

[7] Stone A.L., Wilson A.C. (2016). Transmission of risk from parents with chronic pain to offspring: An integrative conceptual model. Pain.

[8] Wilson A.C., Moss A., Palermo T.M., Fales J.L. (2014). Parent Pain and Catastrophizing Are Associated With Pain, Somatic Symptoms, and Pain-Related Disability Among Early Adolescents. J. Pediatr. Psychol..

[9] Claar R.L., Simons L.E., Logan D.E. (2008). Parental response to children’s pain: The moderating impact of children’s emotional distress on symptoms and disability. Pain.

[10] Pantaleao A., DiPlacido J., Guite J.W., Zempsky W.T. (2019). Caregiver factors related to emergency department utilization for youth with sickle cell disease. Children’s Health Care.

[11] Guite J.W., Kim S., Chen C.-P., Sherker J.L., Sherry D.D., Rose J.B., Hwang W.-T. (2014). Treatment expectations among adolescents with chronic musculoskeletal pain and their parents before an initial pain clinic evaluation. Clin. J. Pain.

[12] Tian F., Guittar P., Moore-Clingenpeel M., Higgins G., Ardoin S.P., Spencer C.H., Jones K., Thomas B., Akoghlanian S., Bout-Tabaku S. (2018). Healthcare Use Patterns and Economic Burden of Chronic Musculoskeletal Pain in Children before Diagnosis. J. Pediatr..

[13] Sieberg C.B., Smith A., White M., Manganella J., Sethna N., Logan D.E. (2017). Changes in Maternal and Paternal Pain-Related Attitudes, Behaviors, and Perceptions across Pediatric Pain Rehabilitation Treatment: A Multilevel Modeling Approach. J. Pediatr. Psychol..

[14] Weiss K.E., Junghans-Rutelonis A.N., Aaron R.V., Harbeck-Weber C., McTate E., Luedtke C., Bruce B.K. (2019). Improving distress and behaviors for parents of adolescents with chronic pain enrolled in an intensive interdisciplinary pain program. Clin. J. Pain.

[15] Kemani M.K., Kanstrup M., Jordan A., Caes L., Gauntlett-Gilbert J. (2018). Evaluation of an Intensive Interdisciplinary pain treatment based on acceptance and commitment therapy for adolescents with chronic pain and their parents: A nonrandomized clinical trial. J. Pediatr. Psychol..

[16] Pielech M., Wallace D.P., Fitzgerald M., Hoffart C.M. (2018). Parent Responses to Child Pain During Intensive Interdisciplinary Pain Treatment and 1-Year Follow-Up. J. Pain.

[17] Wallace D.P., Woodford B., Connelly M. (2016). Promoting psychological flexibility in parents of adolescents with chronic pain: Pilot study of an 8-week group intervention. Clin. Pract. Pediatr. Psychol..

[18] Law E.F., Wan Tham S., Aaron R.V., Dudeney J., Palermo T.M. (2018). Hybrid cognitive-behavioral therapy intervention for adolescents with co-occurring migraine and insomnia: A single-arm pilot trial. Headache.

[19] Guite J.W., Russell B.S., Homan K.J., Tepe R.M., Williams S.E. (2018). Parenting in the Context of Children’s Chronic Pain: Balancing Care and Burden. Children.

[20] Russell B., Guite J. (2019). Parenting impacts from a mindfulness-based pilot intervention for families facing pediatric chronic pain. J. Child. Fam. Stud..

[21] Guite J.W., Russell B.S., Pantaleao A., Thompson Heller A., Donohue E., Galica V., Zempsky W.T., Ohannessian C.M. (2018). Parents as coping coaches for adolescents with chronic pain: A single-arm pilot feasibility trial of a brief, group-based, cognitive–behavioral intervention promoting caregiver self-regulation. Clin. Pract. Pediatr. Psychol..

[22] Walker L.S., Greene J.W. (1991). The functional disability inventory: Measuring a neglected dimension of child health status. J. Pediatr. Psychol..

[23] Reid G.J., Gilbert C.A., McGrath P.J. (1998). The Pain Coping Questionnaire: Preliminary validation. Pain.

[24] von Baeyer C.L., Spagrud L.J., McCormick J.C., Choo E., Neville K., Connelly M.A. (2009). Three new datasets supporting use of the Numerical Rating Scale (NRS-11) for children’s self-reports of pain intensity. Pain.

[25] Baumrind D., Brooks-Gunn J., Lerner R.M., Petersen A.C. (1991). Parenting styles and adolescent development. The Encyclopedia on Adolescence.

[26] Estlein R. (2016). Parenting Styles. Encyclopedia of Family Studies.

[27] Steinberg L. (2001). We know some things: Parent–adolescent relationships in retrospect and prospect. J. Res. Adolesc..

[28] Steinberg L., Silk J. (2002). Parenting adolescents. Handbook of Parenting.

[29] Bowen M. (1993). Family Therapy in Clinical Practice.

[30] Jordan A., Noel M., Caes L., Connell H., Gauntlett-Gilbert J. (2018). A developmental arrest? Interruption and identity in adolescent chronic pain. Pain Rep..

[31] Salamon K.S., Cullinan C.C. (2019). The integrated prevention model of pain—Chronic pain prevention in the primary care setting. Clin. Pract. Pediatr. Psychol..

[32] Palermo T.M., Law E.F., Fales J., Bromberg M.H., Jessen-Fiddick T., Tai G. (2016). Internet-delivered cognitive-behavioral treatment for adolescents with chronic pain and their parents: A randomized controlled multicenter trial. Pain.

[33] Seidman L.C., Martin S.R., Trant M.W., Payne L.A., Zeltzer L.K., Cousineau T.M., Donovan E. (2019). Feasibility and Acceptance Testing of a Mobile Application Providing Psychosocial Support for Parents of Children and Adolescents With Chronic Pain: Results of a Nonrandomized Trial. J. Pediatr. Psychol..

[34] Palermo T.M., de la Vega R., Dudeney J., Murray C., Law E. (2018). Mobile health intervention for self-management of adolescent chronic pain (WebMAP mobile): Protocol for a hybrid effectiveness-implementation cluster randomized controlled trial. Contemp Clin. Trials.

